# Soluble human leukocyte antigen -G during pregnancy and infancy in Benin: Mother/child resemblance and association with the risk of malaria infection and low birth weight

**DOI:** 10.1371/journal.pone.0171117

**Published:** 2017-02-06

**Authors:** Tania C. d’Almeida, Ibrahim Sadissou, Jacqueline Milet, Gilles Cottrell, Amandine Mondière, Euripide Avokpaho, Laure Gineau, Audrey Sabbagh, Achille Massougbodji, Kabirou Moutairou, Eduardo A. Donadi, Benoit Favier, Edgardo Carosella, Philippe Moreau, Nathalie Rouas-Freiss, David Courtin, André Garcia

**Affiliations:** 1 Université Pierre et Marie Curie, Paris, France; 2 UMR 216-MERIT, Institut de Recherche pour le Développement, Faculté de Pharmacie - Université Paris Descartes, Sorbonne Paris-Cité, Paris, France; 3 Centre d’Etude et de Recherche sur le Paludisme Associé à la Grossesse et à l’Enfance, Faculté des Sciences de la Santé, Cotonou, Bénin; 4 Université d’Abomey-Calavi, Cotonou, Bénin; 5 Division of Clinical Immunology, School of Medicine of Ribeirão Preto, University of São Paulo, Brazil; 6 Université Paris Descartes, Sorbonne Paris Cité, Paris, France; 7 UMR 216-MERIT, Institut de Recherche pour le Développement, Campus de la Faculté des Sciences de la Santé (FSS) et de l’Institut des Sciences Biomédicales Appliquées (ISBA), Cotonou, Bénin; 8 Ecole Pasteur – CNAM de santé publique, Paris, France; 9 CEA, Institut des Maladies Emergentes et des Thérapies Innovantes (IMETI), Service de Recherches en Hémato-Immunologie (SRHI), Hôpital Saint-Louis, IUH, Paris, France; 10 Université Paris Diderot, Sorbonne Paris Cité, IUH, Hôpital Saint-Louis, UMR_E5, IUH, Paris, France; Universite de Limoges, FRANCE

## Abstract

Human leukocyte antigen (HLA) G is a tolerogenic molecule involved in the maternal-fetal immune tolerance phenomenon. Its expression during some infectious diseases leading to immune evasion has been established. A first study conducted in Benin has shown that the production of soluble HLA-G (sHLA-G) during the first months of life is strongly correlated with the maternal level at delivery and associated with low birth weight and malaria. However sHLA-G measurements during pregnancy were not available for mothers and furthermore, to date the evolution of sHLA-G in pregnancy is not documented in African populations. To extend these previous findings, between January 2010 and June 2013, 400 pregnant women of a malaria preventive trial and their newborns were followed up in Benin until the age of 2 years. Soluble HLA-G was measured 3 times during pregnancy and repeatedly during the 2 years follow-up to explore how sHLA-G evolved and the factors associated. During pregnancy, plasma levels of sHLA-G remained stable and increased significantly at delivery (*p*<0.001). Multigravid women seemed to have the highest levels (*p* = 0.039). In infants, the level was highest in cord blood and decreased before stabilizing after 18 months (*p*<0.001). For children, a high level of sHLA-G was associated with malaria infection during the follow-up (*p* = 0.02) and low birth weight (*p* = 0.06). The mean level of sHLA-G during infancy was strongly correlated with the mother’s level during pregnancy (<0.001), and not only at delivery. Moreover, mothers with placental malaria infection had a higher probability of giving birth to a child with a high level of sHLA-g (*p* = 0.006). High sHLA-G levels during pregnancy might be associated with immune tolerance related to placental malaria. Further studies are needed but this study provides a first insight concerning the potential role of sHLA-G as a biomarker of weakness for newborns and infants.

## Introduction

Human leukocyte antigen-G (HLA-G) is a nonclassical HLA class I antigen that differs from the others HLA class I molecules in its limited polymorphism, its restricted tissue distribution and its particular expression [1]. Alternative splicing encodes four membrane-bound and three soluble isoforms. The main isoforms present in the plasma are soluble HLA-G1 and -G5 [2]. HLA-G also differs from the HLA class I molecule by exerting inhibitory functions on immune responses [3–5].

The expression of HLA-G during pregnancy and its importance in maternal—fetal tolerance is well established [6, 7]. Indeed, the major physiological role of HLA-G is the protection of the fetal semi-allograft against lysis by maternal NK cells. Moreover, interaction between HLA-G and CD158d in NK cells seems to promote vascularization in maternal decidua during early pregnancy which is important for the development of the fetus [8, 9]. The levels of soluble HLA-G (sHLA-G) are significantly higher in pregnant than in nonpregnant women [10–12]. sHLA-G has been used as a reliable marker for following *in vitro* fertilization [13, 14] and was significantly lower in preeclampsia than in normal pregnancy [15]. In newborns, studies have shown that the levels in cord blood are lower than in mothers [12, 16].

The origin of the variability of sHLA-G blood levels is complex and not fully known, involving genetic mechanisms [17]. Until recently, African populations have been underrepresented in studies despite the greater genetic diversity and heterogeneity observed across Africa [18]. There are currently no data on how sHLA-G evolves during pregnancy in African populations except one study with data available only at delivery for mothers. A strong positive correlation between sHLA-G levels in mothers at delivery and children has been highlighted, and high levels of sHLA-G in children appeared to increase the malaria risk and are associated with low birth weight (LBW) [19], which is highly correlated with neonatal morbidity and mortality [20, 21]. Unfortunately, gestational information was not available in this first study and it seemed important to account for the course of pregnancy.

In case of malaria infection of the placenta an immune tolerance phenomenon has been described as responsible for the higher susceptibility of newborns to malaria infection during the first months of life [22–24]. Due to the important role of HLA-G in immune regulation and immune tolerance and to its strong expression during pregnancy, the potential association between sHLA-G during the whole pregnancy, and not only at delivery and malaria needs to be explored.

In the current study involving pregnant women, our aim was to explore the mother/child resemblance and to confirm or not the association between malaria, LBW and sHLA-G level.

## Materials and methods

### Study design and follow-up

The present follow-up is part of a study concerning 1,183 human immunodeficiency virus (HIV)-negative pregnant women participating in Malaria in Pregnancy Preventive Alternative Drugs (http://clinicaltrials.gov/ct2/show/NCT00811421), a randomized trial of intermittent preventive treatment (IPTp) with either sulfadoxine-pyrimethamine or mefloquine. The first 400 infants born to these women were enrolled from January 2010 to June 2011 and followed throughout the first 2 years of life [25, 26]. Twin pregnancies, stillbirth, or fetal abnormalities were excluded (<1%). In the same way, women with renal, hepatic, neurologic and psychiatric active diseases were not selected. Women were included before the end of 28 gestational weeks (GW) and two doses of IPTp were administered at antenatal visits (ANVs). Between ANVs, women had to attend the health centre for all health complaints. Women were examined and a questionnaire completed. At inclusion, sociodemographic information, socioeconomic characteristics, reproductive and medical history were collected.

Three blood samples were collected during scheduled ANVs (before IPTp) for plasma sHLA-G measurement and malaria diagnosis. After delivery, a placental blood smear was used to assess placental malaria. Malaria infection was defined as the presence of asexual *Plasmodium falciparum* parasites in a blood smear.

Newborns were followed from birth to 24 months. At 6, 9, 12, 18 and 24 months, children were visited and clinically examined, and anthropometric data were collected. Children were regularly visited, a medical questionnaire was filled out and a systematic thick blood smear (TBS) was performed to search for asymptomatic malaria. Mothers were invited to visit health centres with their child if there was any health problem and in case of fever or history of fever, a rapid diagnosis test (RDT) was performed. A malaria attack was defined as an axilary temperature greater or equal to 37.5 (or history of fever) and a positive RDT. Cord blood was sampled at birth, peripheral blood at 6, 9, 12, 18 and 24 months, and the same tests as in mothers were performed. All the medications prescribed were free of charge.

### Soluble HLA-G quantification

Soluble HLA-G was quantified using MEM-G/9 [27], which recognizes the most abundant soluble isoforms (sHLA-G1, -G5) and anti-human β2-microglobulin as capture and detection antibodies, respectively [28]. All incubation steps were performed at room temperature and followed by four washes using washing buffer (H2O, PBS 1X, 0.1% Tween20). The plates were incubated for 30 min with the substrate (Tetramethyl benzidine, Sigma Aldrich, USA) and absorbance was measured at 450 nm after adding HCL (1 N). Total sHLA-G levels were determined from a five-point standard curve (12.5–200 ng/ml) using dilutions of calibrated HLA-G5 purified from M8-HLA-G5 cell line culture supernatant, and the results were expressed as ng/ml. The limit of detection is ~1 ng/ml. The methodology to measure HLA-G using ELISA has been validated [27].

### Ethics

The study protocol and informed consent were approved by the Comité Consultatif de Déontologie et d'Éthique (CCDE) of the Institut de Recherche pour le Développement (IRD, France) and the Ethics Committee of the Faculté des Sciences de la Santé de Cotonou (Benin), the local ethic review Committee. Before inclusion, the study was explained in the local language. A verbal agreement and a written consent were obtained: a copy of this document was given to each participant of the study. In the case that the woman could not read, an impartial witness was involved in the process. In addition to the assent of minors, consent was obtained from the parents or legal guardians. Women were free to interrupt their participation at any time.

### Definition of variables

The outcome variable was the level of sHLA-G in mothers and infants, used as a quantitative variable. To study mother/child resemblance, the mean level of sHLA-G was calculated throughout the pregnancy for women and during follow-up for infants. The cord blood level was not included in the mean calculation. Every woman/infant pair was classified as belonging to one of the four quartiles of the mean distribution depending on their mean level (ng/ml). The quartiles allowed us to define the sHLA-G profile for each individual. For mothers the profiles (P) were: very low (P1≤1.19), low (1.19<P2≤9.11), medium high (9.11<P3≤19.05) and high (19.05<P4); for children: very low (P1≤1.34), low (1.34<P2≤7.21), medium high (7.21<P3≤18.43) and high (18.43<P4).

The following covariates were used:

- For mothers: women’s age, ethnicity (Aïzo, Fon, and others), newborn’s gender and birth weight, parity, GW, placental and peripheral malaria infection during follow-up;
- For children: birth weight, prematurity (≤37 GW), age, gender, height, ethnicity, fever, CRP (C-reactive protein), maternal age, parity, maternal malaria (placental or peripheral) and malaria infection of children.

### Statistical analysis

Data were analyzed with Stata^®^ Software, Version 12 (StatCorp LP, College Station, TX, USA). Mothers and children were analyzed separately.

For mothers, the three sHLA-G measurements were not considered as classical repeated measurements (IPTp administration for the first two ANVs and delivery at the third visit). Different arguments could explain this choice. Pregnancy could be considered as a special period for the mother, related to the presence of the fetus. The first and second antenatal visits (ANV1 and 2) occurred at different times for the women and were characterized by many important interventions of the care team. Therefore, the effects of covariates on the sHLA-G level at each visit were explored separately using a Tobit regression model, a censored regression model, to account for the truncated values generated by the sHLA-G detection limit [29, 30]. The evolving sHLA-G level at each gestational month was compared using a Kruskal-Wallis test with Bonferroni correction. For children, systematic scheduled visits are not independent and the longitudinal data collected along the first two years of children presented a hierarchical two-level structure, where sHLA-G measurements (level 1) were clustered within children (level 2). Hierarchical mixed Tobit regression models were used for univariate and multivariate analyses with an independent variance-covariance structure of the random effects which is the default option [31]. Lastly, mother/child resemblance was explored using the sHLA-G profiles as outcome. Since the profiles were ordered from “very low” to “very high”, an ordinal logistic regression model was used.

All factors with a *p*-value <0.20 during univariate analyses and other interesting factors were included in the multivariate step. Statistical significance was set at *p*<0.05.

## Results

The women’s mean age was 25.9 years (95% confidence interval [25.4–26.4]; range, 18–42). The first ANV occurred before 29 GW and 15.7% were primigravid. Throughout the follow-up, 27.7% of women developed peripheral malaria and 10.9% had placental malaria at delivery.

In children, the mean birthweight was 3033.92 g (IC95%: [2992.50–3075.35]), 9.05% of them had a LBW, and 14 newborns were preterm. During the study, 12.16% of children developed a malaria infection between 6 and 24 months. There is no infection at birth in newborns.

The general characteristics of the study population are presented in Table 1.

**Table 1:** Characteristics of women and newborns in Allada, 2007–2010.

Mothers (*n* = 400)			Newborns (*n* = 400)		
Age		25.92 years	Birth weight		3033.92 g
		SD: 5.45			SD: 420.37
Schooling		29.75% (117)	Low birthweight		9.05% (36)
Ethnicity	Aïzo	69.50% (278)	Prematurity		10.00% (40)
	Fon	20.75% (83)	Gender	Female	53.00% (212)
	Others	9.75% (39)		Male	47.00% (188)
Previous pregnancies	0	15.75% (63)	HLA-G (ng/ml) ^(c)^	Birth	20.16, SD: 28.13
	1	20.75% (83)		6 months	18.17, SD: 33.76
	2	14.75% (59)		9 months	15.60, SD: 35.87
	3	17.25% (69)		12 months	11.23, SD: 19.84
	4	10.00% (40)		18 months	8.38, SD: 15.43
	≥5	21.50% (86)		24 months	9.66, SD: 23.85
Married		97.75% (391)			
Primigravidae		15.75% (63)			
IPTp ^(a)^	SP	34.50% (138)			
	MQFD	35.50% (142)			
	MFSD	30.00% (120)			
Placental malaria		10.75% (43)			
Health center	Attogon	24.00% (96)			
	Sekou	76.00% (304)			
HLA-G (ng/ml) ^(b)^	ANV1	10.08, SD: 13.60			
	ANV2	10.57, SD: 14.05			
	Delivery	17.25, SD: 34.55			

^(a)^ Two molecules were used for IPTp according to the protocol of the MIPPAD study: Sulfadoxine-pyrimethamine (SP: 1500/75 mg) and Mefloquine (MQ: 15 mg/kg), which is given once as a full dose (MQFD) or split over 2 days (MQSD).

^(b)^: mean level of soluble HLA-G in the population at each ANV and at delivery.

^(c)^: mean level of soluble HLA-G in children at birth and at each visit.

### Evolution of soluble HLA-G in pregnancy

**Fig 1:**
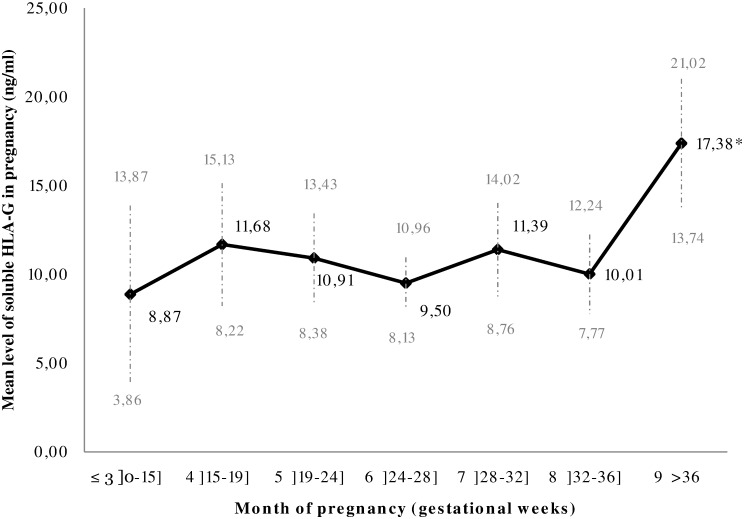
Evolution of the mean level (and 95 confidence intervals) of soluble HLA-G during pregnancy. ^(^*^)^ indicates that the level of sHLA-G is significantly higher at delivery (p<0.001; Kruskal-Wallis test). (≤3 months: n = 26, SD: 12.39, 4 months: n = 65, SD: 13.92, 5 months: n = 177, SD: 17.01, 6 months: n = 254, SD: 11.48, 7 months: n = 131, SD: 15.20, 8 months: n = 115, SD: 12.09, 9 months: n = 360, SD: 35.11).

Blood was sampled in 85% of mothers for the three ANVs, and 98% were sampled at least twice. sHLA-G was described by month of pregnancy after grouping the first 3 months (Fig 1). The sHLA-G level remained stable during pregnancy and increased significantly at delivery (*p*<0.001).

At the first ANV, univariate analysis showed that multigravid women had a higher sHLA-G level (*p* = 0.036) than primigravid women. The sHLA-G level seemed lower in women who took mefloquine for IPTp (*p* = 0.05) and those with placental malaria (*p* = 0.04). In contrast, there is no effect of age, peripheral malaria, foetus gender and LBW. In multivariate analysis, gravidity and IPTp remained significant (*p* = 0.039 and *p* = 0.05, respectively), but the association of sHLA-G with placental malaria became marginally significant (*p* = 0.07).

At the second ANV, previous associations were no longer significant. However, the oldest women (>25 years) had a higher sHLA-G level than the youngest (*p* = 0.009) (Table 2).

**Table 2:** Factors associated with sHLA-G level in pregnancy: Regression of Tobit (univariate and multivariate).

Covariates		ANV 1 (IPT1)				ANV 2 (IPT2)		Delivery			
		β	[95% CI]	Adjus-ted β	[95% CI]	β	[95% CI]	β	[95% CI]	Adjus-ted ß	[95% CI]
			(*p*)		(*p*)		(*p*)		(*p*)		(*p*)
Age (years)	≤ 25	0	[-0.79, 7.34]			0	**[1.70, 10.01]**	0	[-9.93, 8.04]		
	> 25	3.27	(0.11)			5.86	**(0.006)**	−0.95	(0.84)		
Ethnicity	Fon	0	[-5.81, 4.07]			0	[-6.33, 3.83]	0	[-11.36, 10.71]		
	Others	−0.87	(0.73)			−1.25	(0.63)	−0.32	(0.95)		
Gender	Male	0	[-5.16, 2.96]			0	[-5.90, 2.44]	0	[-11.12, 6.81]		
	Female	−1.09	(0.59)			−1.73	(0.41)	−2.15	(0.64)		
Primigravidity	No	0	**[-11.94, -0.41]**	0	**[-11.79, -0.30]**	0	[-8.98, 2.65]	0	[-13.96, 11.12]		
	Yes	−6.17	**(0.036)**	−6.04	**(0.039)**	−3.17	(0.28)	−1.42	(0.82)		
IPT	SP + MQFD	0	**[-8.87, 0.09]**	0	**[-8.75, 0.18]**	0	[-1.38, 1.75]	0	[-6.49, 13.02] (		
	MQSD	−4.38	**(0.05)**	−4.4	**(0.05)**	−2.81	(0.23)	3.26	0.511)		
Placental infection	No	0	**[-13.65, -0.18]**	0	[-12.93, 0.61]	0	[-11.59, 2.02]	0	[-27.59, 1.66]	0	[-28.12, 1.14]
	Yes	−6.92	**(0.04)**	−6.16	(0.07)	−4.78	(0.17)	−13.0	(0.08)	−13.49	(0.07)
Peripheral malaria	No	0	[-4.26, 6.76]	0		0	[-12.17, 8.32]	0	[-22.65, 6.18]		
	Yes	1.25	(0.67)	2.87		−1.92	(0.71)	−8.23	(0.26)		
LBW	No	0	[-1.17, 9.36]	0		0	[-12.60, 2.44]	0	[-2.52, 28.42]	0	[-1.94, 29.02]
	Yes	4.09	(0.13)	−4.25		−5.08	(0.18)	12.95	(0.10)	13.54	(0.086)

At delivery, there was no significant association. Women giving birth to a LBW newborn had a higher sHLA-G level and women with placental malaria had lower levels, but the differences were marginally significant (*p* = 0.086 and *p* = 0.07, respectively) (Table 2).

### Soluble HLA-G during infancy

**Fig 2:**
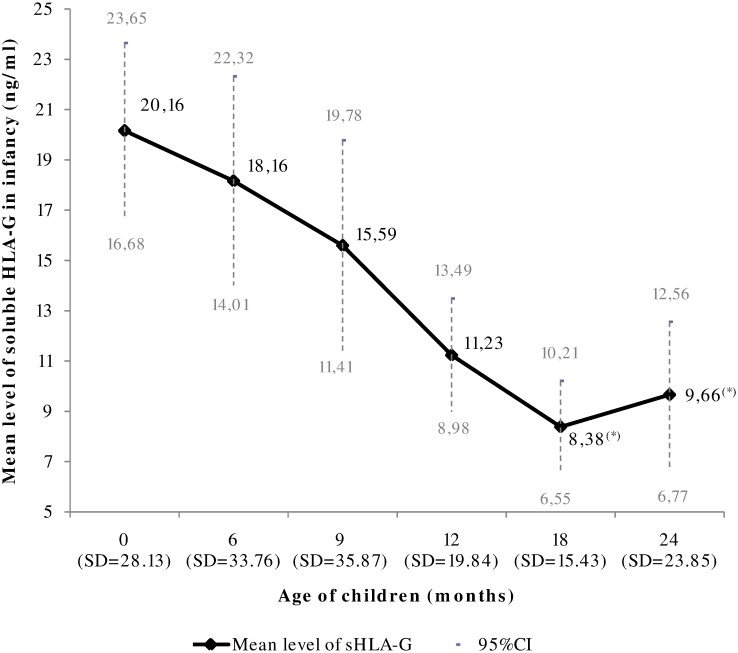
Evolution of the mean level of soluble HLA-G in the first two years of life (p<0.001 for the overall evolution; mixed Tobit model). ^(^*^)^ The mean level of soluble HLA-G in infants at 18 and 24 months are not significantly different (p = 0.77).

Blood was collected for 373 newborns, 311 with at least three samples. The level was highest in cord blood, and decreased until 18 months (*p*<0.001) (Fig 2).

**Fig 3:**
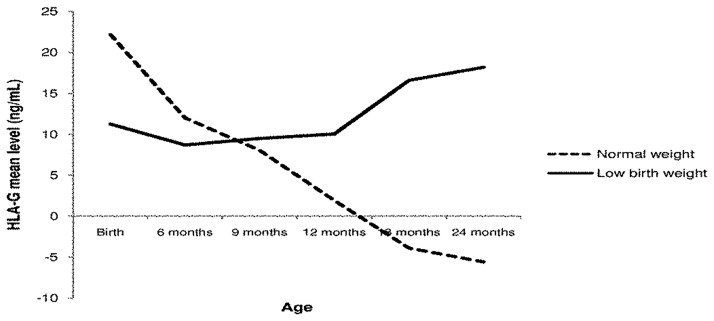
Predicted evolution of sHLA-G from birth to 24 months for low birth weight versus normal birth weight children.

There was no significant association between gender, birth weight and sHLA-G level. Mothers’ malaria infection (placental or peripheral) was not associated with the children’s sHLA-G level. In contrast, children infected by malaria at least once during the follow-up had a higher sHLA-G level (*p* = 0.02) (Table 3). We identified an interaction between age and LBW showing that children with LBW had higher sHLA-G levels and a different evolution during the first 24 months of life (*p* = 0.06) (Fig 3).

**Table 3:** Factors associated with sHLA-G level in infancy: Regression of Tobit (univariate and multivariate mixed models).

Covariates		β	[95% CI]	Adjusted β^(a)^	[95% CI]
			(*p)*		*(p)*
Gender	M	0	[-4.05, 8.17]		
	F	2.06	(0.51)		
LBW	No	0	[-5.22, 17.23]	0	[-29.72, 7.88]
	Yes	6.001	(0.29)	−10.92	(0.25)
Fever	No	0	[-6.07, 5.91]		
	Yes	−0.08	(0.98)		
Malaria infection	No	0	**[0.76, 14.14]**	0	[**1.28, 14.68**]
	Yes	7.45	**(0.03)**	7.92	**(0.02)**
Positive CRP	No	0	[-2.86, 7.39]		
	Yes	2.26	(0.39)		
Maternal age	≤ 25 years	0	[-2.90, 9.36]		
	> 25 years	3.23	(0.31)		
Primigravidity	No	0	[-11.93, 4.98]		
	Yes	−3.47	(0.42)		
Ethnicity	Fon	0	[-7.64, 7.23]		
	Others	−0.2	(0.96)		
Placental malaria	No	0	[-7.48, 11.83]		
	Yes	2.17	(0.66)		
Age **(months)**	0	0		0	
	6	−8.67	**[-16.85, -3.48]**	−10.16	[**-16.85, -3.48**]
	9	−11.69	**[-20.71, -7.53]**	−14.12	[**-20.71, -7.53**]
	12	−17.65	**[-26.86, -13.64]**	−20.25	[**-26.86, -13.64**]
	18	−23.07	**[-33.03, -19.11]**	−26.07	[**-33.03, -19.11**]
	24	−22.27	**[-34.83, -20.68]**	−27.75	[**-34.83, -20.68**]
			**(<0.001)**		**(<0.001)**
LBW*Age **(months)** (interaction)	0			0	
	6			7.6	**[-17.48, 32.70]**
	9			12.36	**[-11.73, 36.45]**
	12			19.04	**[-4.60, 42.68]**
	18			31.39	**[5.44, 57.34]**
	24			34.68	**[9.40, 59.97]**
					**(0.06)**^(b)^

^(a)^ The final multivariate model contains LBW, malaria infection, age and interaction LBW*Age.

^(b)^ For the interaction LBW*Age, p = 0.06 represents the p global, and shows that the evolution of soluble HLA-G level is variable according to the birth weight: overall, the mean level of soluble HLA-G is higher in case of LBW, while the mean level in newborns with normal weight seems lower. This difference is more significant at 18 (p = 0.05) and 24 months (p = 0.03).

### Mother/child resemblance

As described above, the sHLA-G level in children is correlated with their mother’s level. There were a strong correlation (*p*<0.001) between each mother’s cross-sectional measurement and her child’s levels throughout the follow-up (Table 4) and an association between the mother’s profile and her child’s mean sHLA-G level during the follow-up (*p*<0.001). Children born to a mother with very low profile had the lowest level, whereas children born to a mother with very high profile had the highest mean sHLA-G level.

**Table 4:** Correlation between mothers’ and children’s levels of soluble HLA-G (Spearman’s rho).

Spearman’s rho (*p*)	Cord blood	6 months	9 months	12 months	18 months	24 months
ANV1	0.34 (<*0*.*001*)	0.44 (<*0*.*001*)	0.40 (<*0*.*001*)	0.48 (<*0*.*001*)	0.46 (<*0*.*001*)	0.48 (<*0*.*001*)
ANV2	0.37 (<*0*.*001*)	0.36 (<*0*.*001*)	0.38 (<*0*.*001*)	0.41 (<*0*.*001*)	0.39 (<*0*.*001*)	0.50 (<*0*.*001*)
Delivery	0.39 (<*0*.*001*)	0.35 (<*0*.*001*)	0.36 (<*0*.*001*)	0.39 (<*0*.*001*)	0.39 (<*0*.*001*)	0.39 (<*0*.*001*)

**Fig 4:**
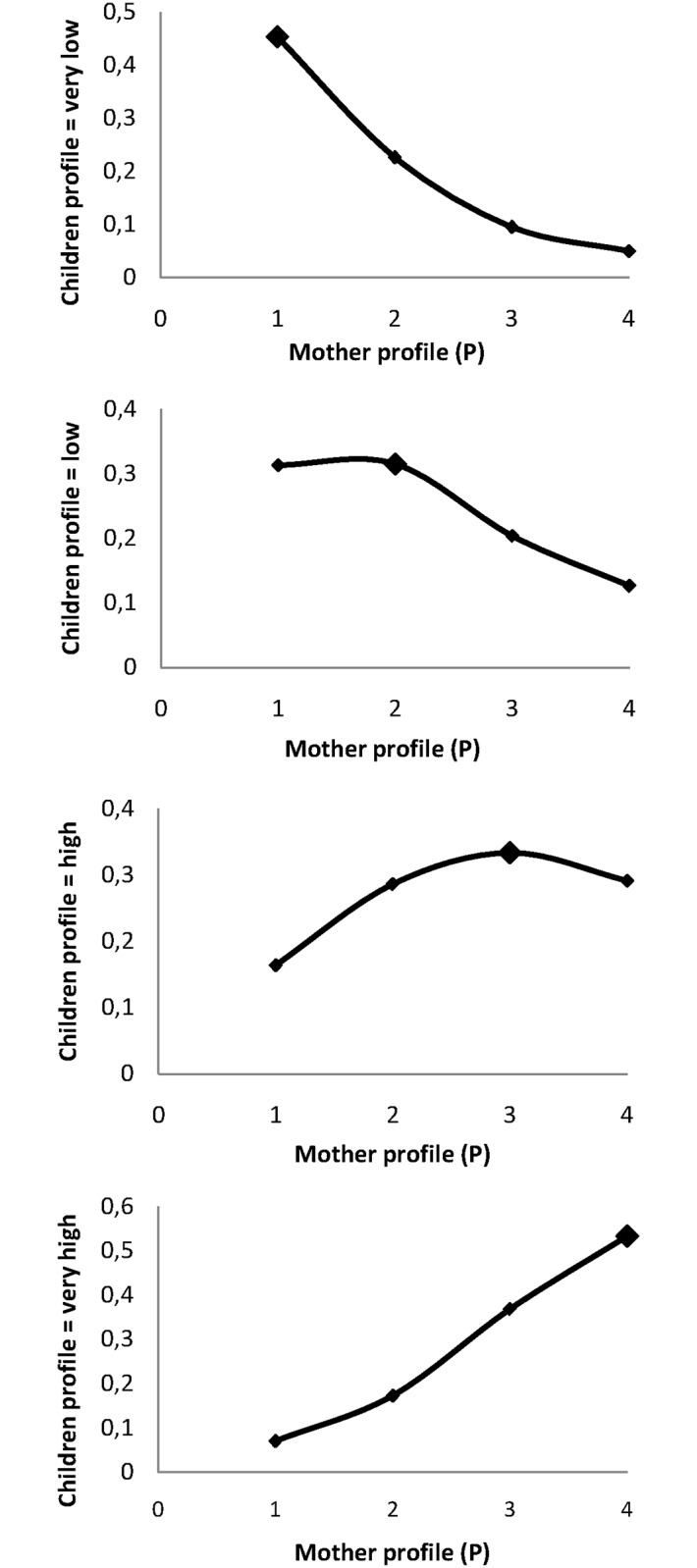
Mother/child resemblance: Probability of newborn to have a profile according to the mother profile.

Using multinomial ordinal models, a mother belonging to a certain profile had a significantly higher probability of giving birth to a child belonging to the same profile (Fig 4). Furthermore, the odds ratio for giving birth to a child with a “very high” profile versus all others was strongly associated with the increase in the mother’s profile (*p*<0.001). For an increase in the mother’s profile from “very low” to “low”, this OR was 3.05 (CI95 = [1.75–5.31]). The OR was 17.59 (CI95 = [9.54–32.41]) for an increase from “very low” to “very high” (Table 5). The OR for giving birth to a child with a very high profile for a mother with placental malaria, adjusted on the mother’s profile, was 2.39 [1.29–4.45] compared to a mother without placental infection (Table 5).

**Table 5:** Maternal factors associated with the probability to belong to a high sHLA-G profile in infancy: Ordinal multinomial regression.

Variable		OR^(a)^	95 confidence interval	*p*
**Mother’s sHLA-G profile**	Very low	1		
	Low	3.05	[1.75, 5.31]	<0.001
	High	8.28	[4.68, 14.67]	<0.001
	Very high	17.59	[9.54, 32.41]	<0.001
**Placental malaria infection**	No	1		
	Yes	2.39	[1.29, 4.45]	0.006

^**(a)**^ Odds ratio of giving birth to a child with very high profile for a mother with low, high and very high profile compared to a mother with a very low profile.

## Discussion

The present study is the first conducted in an African population describing the physiological evolution of the sHLA-G level during pregnancy. This work complements a study conducted in Benin that found a strong correlation between maternal sHLA-G level at delivery and children’s levels during the first years of life as well as an association between the infant’s level and LBW and malaria [19]. However, in this study mothers were included at delivery and pregnancy data were not available. Associations between both malaria and LBW and the infant’s sHLA-G level were confirmed here. Furthermore, the correlation between the mother’s and the child’s sHLA-G levels was reinforced since we showed that the child’s level throughout the follow-up was correlated with its mother’s level at each measurement. Finally, mothers with placental malaria gave birth to children with high sHLA-G profile during infancy. This last result is consistent with the fact that the immune tolerance described in case of placental malaria [22–24] could be correlated with sHLA-G levels and that placental malaria might be considered as a marker of a more complex phenomenon involving the tolerogenic molecule HLA-G.

The analysis of how the sHLA-G level evolved showed that it remained stable but increased considerably at delivery. In different populations, Hunt *et al*. and Klitkou *et al*. found that the sHLA-G level increased in early pregnancy and decreased later [12, 16]. In the first study, the majority of samples were collected during the first trimester and differences in levels during the first, second and third trimesters were not significant [12]. Moreover, only 22 women out of 129 were sampled more than once. In the second study, sHLA-G levels were significantly higher at 20 GW than at term, after 37 GW, without indicating the time between the last sampling and delivery [16]. Another study concluded that sHLA-G level is constant with a peak in the third month [10]. However, in this study the sHLA-G levels of pregnant women were compared to non-pregnant women. Nevertheless, the main difference between these previous analyses and our results comes from the population and its behaviour. It has been described that in rural Africa pregnant women reach the maternity clinic late, and most often labour is already advanced [32]. Yet, Knafel *et al*. showed that the progression of labour is associated with a continuous and significant increase in the sHLA-G plasma level [33]. In 2009, Rizzo et *al* evaluated the effect of labour on the plasma sHLA-G levels by measuring sHLA-G5 in 43 women at 3 times: during the 3^rd^ trimester, at labour and at 2 years postpartum. They found that sHLA-G was significantly increased during labour in comparison with the others times [34]. The elevation of the protein could be associated with the stress induced by labour and be part of a cascade reaction leading to foetal expulsion. Another important difference is the genetic origin of the population. It is now accepted that African populations differ from Caucasian populations in their greater genetic diversity and heterogeneity [18], and these differences could influence the variability of sHLA-G levels [17, 35, 36]. The particular role of HLA-G in immunity and the competing needs to maintain both maternal and foetal immune tolerance and an efficient host immune response make this gene a potential target for selection. This phenomenon has been recently demonstrated in African populations including a Beninese population, highly exposed to multiple infectious agents [37]. This multiplicity of exposure to various infections represents another potential difference between our population and Caucasian groups.

The three sHLA-G measurements were not considered as classical repeated measurements. The following arguments explain this choice. We are facing a special population, constituted by pregnant women. Pregnancy is a special period for the women, with important physiological changes related to the presence of the fetus, and these changes are variable according to the gestational age and from a woman to another one. In this study, the first and second antenatal visits (ANV1 and 2) occurred at different times for women (several week of lag as shown in Fig 1). Secondly, these ANV were characterized by many important interventions of the care team such as administration of several medicines (intermittent preventive treatment against malaria, antihelminthics, iron and folic acid supplements…). Finally, delivery represents a very particular moment with a possible great variability in women. For these reasons, we have chosen not to consider the measurements performed during pregnancy mothers as classical repeated data and we decided to make transversal analyses.

At the beginning of pregnancy, multigravid women have higher sHLA-G levels than primigravid women and sHLA-G increases gradually with the number of pregnancies (data not shown). It could be that multigravid subjects produce more HLA-G and have a better aptitude for acceptance of semiallogenic foetal tissue. In other studies, a gravidity effect was not found [10, 16], but here again Caucasian and African populations differ since in our study approximately 50% of the women had had at least three pregnancies before the study. The low level of sHLA-G in primigravid women is consistent with the fact that preeclampsia, more prevalent in first-time pregnancies, has been found frequently associated with a decreased level of soluble HLA-G [38]. The difference observed between primi and multigravid mothers was only present at the beginning of pregnancy. Later during pregnancy this association was no more significant. Age and gravidity are classically correlated and the association between sHLA-G and age at the second ANV could be related to this association. Indeed the effect of gravidity could be more important at the beginning of pregnancy to allow the correct implantation of the embryo.

The source of sHLA-G in newborns is not precisely known and documented to date. We hypothesize that this HLA-G could be produced by the organs of newborns himself such as thymus or umbilical cells [39] or immune cells, but also resulted mainly for placental production after a transfer at the end of pregnancy. The protein could stay in the newborn serum during the first three months, and disappear gradually until six months. To date, there is no data about this production of HLA-G in newborns to our knowledge.

Malaria infection (placental or peripheral) in mothers has no effect on the sHLA-G level throughout pregnancy. This might be due to the substantial increase of sHLA-G in pregnancy, which could result in not detecting a malaria effect. It is noteworthy that pregnant women received two supervised doses of Intermittent Preventive Treatment against malaria, as recommended by WHO in all endemic areas, and that both the incidence of clinical malaria and parasitemia were low, for all IPT groups [26]. Independently of this parameter, this result may seem surprising since during pregnancy the presence of HLA-G would be associated with a reduction in the cytotoxicity of NK cells and should result in a decrease in the immunity responsible for placental infection such as in the case of human cytomegalo-virus infection [40]. The difference between these infections could be due to the production of proinflammatory cytokines which could induce interruption of HLA-G cycle more in human CMV infection than in malaria infection. However, in infancy, the association between high levels of sHLA-G and malaria infection was confirmed. It has recently been shown that not only high levels of sHLA-G could increase the risk of developing a malaria attack, but also that the presence of malaria infection at sampling is associated with high sHLA-G levels [19]. During HIV or hepatitis infections, patients have higher sHLA-G levels, and it has been suggested that these levels contribute toimmune evasion of virus by inducing immune tolerance [41, 42]. We could hypothesize that a similar phenomenon occurs during malaria. *P*. *falciparum* could upregulate the HLA-G secretion by stimulating cytokines such as IL-10 and IFN-γ, which are well-known HLA-G inducers. HLA-G inhibits the function of T, NK, dendritic cells, neutrophils and B cells through direct interaction with ILT2 and/or ILT4 receptors [40, 43–47]. Previous reports indicate that HLA-G may have dual role on NK cells depending on its interaction with either KIR2DL4 or ILT2. In this context, the interaction of HLA-G with ILT2 would favour the inhibition NK cell cytolytic function while its interaction with KIR2DL4 may promote vascularization processes [8, 9]. Therefore, the impact of HLA-G on NK cell functions should be considered at two levels since it may have a role in immune responses against malaria but also in vascularization processes induced during pregnancy. Besides its dual role on NK cells, sHLA-G is also a negative regulator of B cells leading to antibody secretion inhibition in a mouse model [48]. It has long been recognized that antibodies play a pivotal role in anti-malarial protection [49]. Consequently, the inhibition of IgG specifically directed against *P*. *falciparum* mediated by HLA-G may allow the parasite to escape the immune system and be responsible for higher susceptibility to infection. We have shown recently that high level of sHLA-G is associated with a higher probability to develop a malaria attack in the following weeks but also that the presence of malaria infection can increase sHLA-G that could lead to immune evasion [19, 50]. Overall, the results underline the complexity of the association between HLA-G and malaria and need further experimental exploration. Furthermore, it is important to notify that in this study, all women included and their children are virus (HIV)-negative. The high level of soluble HLA-G observed in a part of them, could not be due to HIV infection. According to hepatitis, we do not evaluate the incidence of this infection in the study population but mothers who had an evolutive hepatic disease were not included. Moreover, as recommended by the world health organization, all the newborns of the program received different doses of the vaccine against hepatitis B infection, the most prevalent in this region, and they are normally protected. We did not performed virological or bacterial diagnosis tests neither during pregnancy nor during the follow-up of the children. HLA-G has been associated with these viral infections [41, 42] and this could influence our results. However, all the included individuals can be considered as comparable concerning the exposure to viral infections and we do not consider that this risk may introduce a systematic bias.

The association between HLA-G and LBW shows that the evolution of sHLA-G during the first 2 years of life strongly differs between LBW children and others. LBW is correlated with infants’ morbidity and mortality [20, 21] and this result is consistent with our previous finding that some children could have particular trajectory of sHLA-G during infancy [50]. The present result strengthens the hypothesis that the susceptibility of LBW children to infections could be associated with high sHLA-G levels [19], and that infants with LBW could have a particular mechanism of HLA-G regulation.

Our results also confirmed the strong correlation between the mother’s sHLA-G levels at delivery and the cord blood level [19]. Comparable findings were described by Klitkou *et al*., even though the sHLA-G level in cord blood was lower than at term [16]. However, since repeated measurements were available for both women and infants, mother/child resemblance was explored more precisely. It was shown that not only does a mother/child correlation exist at delivery, but also that each mother’s measurement was correlated with each measurement in her child. Moreover, it was also demonstrated that a mother harbouring a sHLA-G profile during pregnancy has a higher probability of giving birth to a child with a similar profile. This kind of resemblance could obviously be due to a shared family environment and behaviour. However, a genetic or epigenetic origin must also be discussed. Indeed, even though the genetic control of sHLA-G secretion is complex [17, 35], it has recently been shown that there is an association between combined feto-maternal HLA-G genotypes and the sHLA-G level [51].

Mother/child resemblance could have consequences from a public health point of view. If it is confirmed that high levels of sHLA-G are associated with LBW and a higher risk for malaria (or more generally for infections), the mother’s sHLA-G level during pregnancy could be a useful biomarker of frailty in children before delivery.

It is accepted that children born from placental malaria are more susceptible to malaria during infancy, suggesting that this could stem from immune tolerance [23, 52, 53]. Such children are not only more susceptible to malaria but also to non-malaria fevers [54], emphasizing the fact that immune tolerance could imply immunity in a more general manner besides specific immunity to malaria. In that sense, placental malaria could just be a biomarker of this more complex phenomenon. These results, showing that mothers with placental malaria have a significant probability of giving birth to a newborn with high levels of sHLA-G during infancy, possibly making the child more susceptible to infections, strongly support this hypothesis and could have important consequences from a public health point of view.
